# Bone marrow stromal cells generate an osteoinductive microenvironment when cultured on titanium–aluminum–vanadium substrates with biomimetic multiscale surface roughness

**DOI:** 10.1088/1748-605X/acbf15

**Published:** 2023-03-08

**Authors:** Michael B Berger, D Joshua Cohen, Kyla B Bosh, Marina Kapitanov, Paul J Slosar, Michael M Levit, Michelle Gallagher, Jeremy J Rawlinson, Zvi Schwartz, Barbara D Boyan

**Affiliations:** 1 Department of Biomedical Engineering, College of Engineering, Virginia Commonwealth University, 601 W. Main Street, Richmond, VA 23284, United States of America; 2 SpineCare Medical Group, 455 Hickey Blvd., Suite 310, Daly City, CA 94015, United States of America; 3 Medtronic, Applied Research—Spine, Minneapolis, MN, United States of America; 4 Department of Periodontology, University of Texas Health Science Center at San Antonio, 7703, Floyd Curl Drive, San Antonio, TX 78229, United States of America; 5 Wallace H. Coulter Department of Biomedical Engineering at the Georgia Institute of Technology and Emory University, 313 Ferst Drive, Atlanta, GA 30332, United States of America

**Keywords:** titanium, osseointegration, nanoroughness, osteoblasts, mouse *in vivo* model, osteoinduction, BMP2

## Abstract

Osseointegration of titanium-based implants possessing complex macroscale/microscale/mesoscale/nanoscale (multiscale) topographies support a direct and functional connection with native bone tissue by promoting recruitment, attachment and osteoblastic differentiation of bone marrow stromal cells (MSCs). Recent studies show that the MSCs on these surfaces produce factors, including bone morphogenetic protein 2 (BMP2) that can cause MSCs not on the surface to undergo osteoblast differentiation, suggesting they may produce an osteogenic environment *in vivo*. This study examined if soluble factors produced by MSCs in contact with titanium–aluminum–vanadium (Ti6Al4V) implants possessing a complex multiscale biomimetic topography are able to induce osteogenesis ectopically. Ti6Al4V disks were grit-blasted and acid-etched to create surfaces possessing macroscale and microscale roughness (MM), micro/meso/nanoscale topography (MN), and macro/micro/meso/nanoscale topography (MMN^TM^). Polyether-ether-ketone (PEEK) disks were also fabricated by machining to medical-grade specifications. Surface properties were assessed by scanning electron microscopy, contact angle, optical profilometry, and x-ray photoelectron spectroscopy. MSCs were cultured in growth media (GM). Proteins and local factors in their conditioned media (CM) were measured on days 4, 8, 10 and 14: osteocalcin, osteopontin, osteoprotegerin, BMP2, BMP4, and cytokines interleukins 6, 4 and 10 (IL6, IL4, and IL10). CM was collected from D14 MSCs on MMN^TM^ and tissue culture polystyrene (TCPS) and lyophilized. Gel capsules containing active demineralized bone matrix (DBM), heat-inactivated DBM (iDBM), and iDBM + MMN-GM were implanted bilaterally in the gastrocnemius of athymic nude mice (*N* = 8 capsules/group). Controls included iDBM + GM; iDBM + TCPS-CM from D5 to D10 MSCs; iDBM + MMN-CM from D5 to D10; and iDBM + rhBMP2 (R&D Systems) at a concentration similar to D5–D10 production of MSCs on MMN^TM^ surfaces. Legs were harvested at 35D. Bone formation was assessed by micro computed tomography and histomorphometry (hematoxylin and eosin staining) with the histology scored according to ASTM 2529–13. DNA was greatest on PEEK at all time points; DNA was lowest on MN at early time points, but increased with time. Cells on PEEK exhibited small changes in differentiation with reduced production of BMP2. Osteoblast differentiation was greatest on the MN and MMN^TM^, reflecting increased production of BMP2 and BMP4. Pro-regenerative cytokines IL4 and IL10 were increased on Ti-based surfaces; IL6 was reduced compared to PEEK. None of the media from TCPS cultures was osteoinductive. However, MMN-CM exhibited increased bone formation compared to iDBM and iDBM + rhBMP2. Furthermore, exogenous rhBMP2 alone, at the concentration found in MMN-CM collected from D5 to D10 cultures, failed to induce new bone, indicating that other factors in the CM play a critical role in that osteoinductive microenvironment. MSCs cultured on MMN^TM^ Ti6Al4V surfaces differentiate and produce an increase in local factors, including BMP2, and the CM from these cultures can induce ectopic bone formation compared to control groups, indicating that the increased bone formation arises from the local response by MSCs to a biomimetic, multiscale surface topography.

## 1. Introduction

Orthopedic and dental implant success is related to the extent of osseointegration around an implanted material. Osseointegration involves a complex biological cascade that occurs immediately after implant placement and encompasses a regenerative immune response to the implanted biomaterial resulting in cellular recruitment, differentiation, and activation of progenitor cells that secrete and mineralize organic matrix, anchoring the implant with native bone tissue [1, 2]. Currently, compromised bone architectures and dysregulation of osseointegration are leading causes of insufficient integration, implant loosening, and ultimate failure [3–7].

Titanium (Ti) and its alloys are often used for implants that interface with bone due to superior material properties, including a naturally occurring passivated oxide layer that prevents material corrosion and provides excellent wear resistance, robust mechanical properties, and tunable surface properties [8, 9]. Polyether-ether-ketone (PEEK) polymers have been investigated for use in bone because they have a higher mechanical modulus than other biocompatible polymers, ranging from 3.6 GPa to upwards of 18 GPa [8], yet lower than metals. Moreover, PEEK is radiolucent, which allows clinicians to view bone growth radiographically. However, PEEK possess dramatically different surface chemistries compared to titanium and its alloys. PEEK-based interbody fusion devices often require the use of recombinant human bone morphogenetic protein-2 (BMP2), particularly when iliac crest bone graft is not available to achieve sufficient fusion [8, 10, 11]. In addition to concerns related to the use of BMP2 and high material costs [12, 13], studies investigating the use of PEEK during osseointegration have observed biological responses that lead to fibrous encapsulation, implant migration, or non-unions [14–17].

Implants fabricated using Ti and its alloys (e.g. titanium–aluminum–vanadium [Ti6Al4V]) have improved biocompatibility compared to PEEK and their ability to support osseointegration has been further improved by modifying their surface topography through a variety of methods to control cellular response [18–20]. Biomimicry can be achieved by creating a surface topography similar to native bone after osteoclastic surface resorption. Surfaces possessing a biomimetic topography have been shown to increase osteoblast progenitor cell differentiation, maturation, and activity *in vitro* and increase implant retention rates *in vivo*. Analysis of these biomimetic surfaces has focused primarily on parameters such as: macroscale roughness, microscale roughness, mesoscale and nanoscale feature formation, surface wettability, and surface and bulk chemical properties [21–24].

Numerous studies have assessed the effects of a variety of nano-topographies on the responses of bone marrow stromal cells (MSCs) to implant surfaces [25–29], and have shown that osteoblastic differentiation is enhanced compared to unmodified surfaces. However, studies assessing the effects of surface parameters on the osteoblastic differentiation of human MSCs indicate that the best results are achieved when there is a multiscale arrangement that includes macro, micro, meso and nanoscale topography. In addition to promoting osteoblast differentiation, these surfaces also result in the production of factors than can control the responses of other cells through paracrine signaling [30–32]. Yet, not all surfaces that possess a complex multiscale topography elicit comparable responses. Studies have demonstrated that a combination of proprietary grit-blasting and acid-etching alter subtle implant surface properties, including kurtosis (pointedness or peakedness) and skewness (symmetry or asymmetry of distribution relative to a bell curve). While surfaces may demonstrate similar levels of average microroughness, *in vitro* models show that cells are able to differentiate between these topographic properties [33].

There is a considerable body of literature demonstrating that MSCs and normal human osteoblasts (NHOsts) will form multi-layered nodules when cultured on tissue culture polystyrene (TCPS) surfaces for 21 d, particularly when they are grown in a culture media containing supplements including dexamethasone and beta-calcium phosphate [34, 35]. In contrast, when these cells are cultured on a Ti6Al4V surface that has a multiscale surface topography that is similar to that of an osteoclast resorption pit, they exhibit osteoblast properties within 7 d, even when using growth media without these supplements [36, 37]. Not only do they produce proteins that are associated with well-differentiated osteoblasts such as osteocalcin (OCN), they also synthesize and secrete proteins that can act on other cells not on the surface, such as BMPs, vascular endothelial growth factor, and transforming growth factor beta-1 (TGF*β*1) needed for osteogenesis [33, 38, 39].

These *in vitro* observations suggest that cells on the biomimetic surface can modulate the local factors in the surrounding environment that influence overall osteogenesis around an implant. This modulation can be further assessed with negative controls. For example, addition of anti-BMP2 antibody to the MSC cultures, as described above, blocks the surface effect on osteoblast differentiation, indicating that BMP2 acts in an autocrine manner. It also blocks production of factors associated with vasculogenesis and modulation of the immune mediators, indicating that the factors produced by MSCs cultured on Ti6Al4V disks with a multiscale biomimetic topography can induce osteoblast differentiation of MSCs on inserts above the surface via paracrine regulation and that BMP2 is one of the regulatory factors involved [40, 41].

These culture experiments strongly support the hypothesis that surface topography can promote osseointegration by inducing MSCs to generate an osteogenic or osteoinductive microenvironment. Testing that hypothesis, however, requires a model to assess bone formation, and *in vitro* models are limited in demonstrating mineralized tissue outcomes. To address this, we adapted an international standard that is recognized by the United States Food and Drug Administration for demonstrating osteoinduction by demineralized bone matrix (DBM) *in vivo*. We reasoned that biomimetic implants themselves are not osteoinductive by the standard definition that bone must form in a site that would otherwise not form bone, such as muscle [42, 43]. Instead, we focused on the microenvironment, represented by the factors produced by cells in response to surface chemistry and topography, and we tested those factors present in the culture media for osteoinductivity using the standard *in vivo* model. We assessed the conditioned media (CM) generated by MSCs cultured on three different multiscale Ti6Al4V surface topographies and machined PEEK for production of factors associated with osteogenesis, and then based on production of BMP2, we analyzed the *in vivo* osteoinduction ability of CM generated by MSCs cultured on a biomimetic surface with macroscale/microscale/mesoscale/nanoscale topography. CM from MSCs cultured on TCPS, media incubated on TCPS without cells, and rhBMP2 at the concentration present in the combined CM from MSCs on the multiscale biomimetic surface as well as active DBM and heat inactivated DBM (iDBM) were used as controls.

## 2. Methods

### 2.1. Substrate preparation

PEEK discs were prepared from 15 mm diameter rods of PEEK bulk material and machined into 1.6 mm thick discs. Titanium–aluminum–vanadium (Ti6Al4V) discs were prepared by Medtronic (Minneapolis, MN) from 15 mm diameter rods of grade 23-alloyed Ti6Al4V machined into 1.6 mm thick disks. A portion of the Ti6Al4V surfaces underwent a grit blasting, chemical masking and dual acid etching process to generate macroscale peaks and valleys creating a patented macroscale/microscale rough Ti6Al4V surface (MM) [44]. A subset of the MM discs received an additional patented proprietary grit-blasting and acid-etching procedure [44–46], to create a complex surface texture at the macro/micro/nanoscale (MMN^TM^). The remaining portion of machined Ti6Al4V also underwent the proprietary grit-blasting and acid-etching procedure to generate a complex surface at the micro/meso/nanoscale (MN) [44, 45, 47]. All discs were cleaned, packaged, and gamma irradiated according to the manufacturer’s protocols.

### 2.2. Surface characterization

#### 2.2.1. Scanning electron microscopy (SEM)

Surface topography was qualitatively assessed using SEM (Hitachi SU-70, Tokyo, Japan). Surfaces were secured on SEM imaging mounts by carbon tape and imaged with 32 μA ion current, 5 kV accelerating voltage and 4 mm working distance. PEEK samples were platinum sputter coated at 1 × 10^−4^ vacuum for 90 s before imaging. Surface homogeneity was determined by viewing multiple locations across the implant surface and three representative locations were imaged at magnifications of 35, 100, 1000, 2000, 10 000, 50 000, and 100 000X to ensure homogenous assessment, with at least two disks per group imaged.

#### 2.2.2. Laser confocal microscopy (LCM)

Surface microroughness was qualitatively assessed by LCM (Zeiss LSM 710). Single plane and Z-stacks were obtained with a Plan Apochromat 20×/0.8 M27 objective with a 5× optical zoom, using a 405 nm laser in reflection mode at 25% power. Scan parameters were 0.79 μs pixel dwell, 0.40 μm pixel size, 600.9 μm pinhole, 202.20 × 202.20 μm image size, and step size of 1 μm. No digital gain was used. Digital bandpass filtering was used: Gaussian 1st order filter with thresholds at 2 μm and 75 μm. Average surface roughness (Sa) was defined as the average absolute distance in the *z*-plane; peak-to-valley distant (Sz) was defined as arithmetic average peak-to-valley height of 25 uniform areas within each field of view calculated within the topography module using ZEN software (Zeiss [48]) and shown as the mean and standard deviation (SD) for *n* = 10 samples from two discs per group.

#### 2.2.3. Sessile drop contact angle

Surface energy was determined by sessile drop test using a goniometer (CAM 250, Ramé-Hart). Samples (*n* = 2) were measured in five different locations and dried with nitrogen between measurements. The 3 µl of ultrapure water was used per drop measurement, and angle measurements were taken every 5 s for a total of 15 s. Those four measurements were then averaged to produce 1 of the 5 total measurements per disc. Measurements are shown as mean ± SD of ten samples per group.

#### 2.2.4. X-ray photoelectron spectroscopy (XPS)

XPS was used to analyze surface chemistry (PHI VersaProbe III Scanning XPS, Physical Electronics Inc., Chanhassen, MN). Copper clips and instrument mount were sonicated in ethanol for 10 min prior to securing samples. Analysis was conducted using a 25 watt, 50 kV x-ray gun with a spot size of 200 µm, 20 ms dwelling time and 1 eV step size. Survey scan was taken at 280 000 eV scan resolution, region scans were taken at 55 000 eV for six elements: oxygen, carbon, titanium, aluminum, vanadium, and nitrogen. Five locations were analyzed per discs with two discs per group. Measurements are shown as mean ± SD of ten samples per group.

#### 2.2.5. X-ray diffraction spectroscopy (XRD)

XRD was used to analyze bulk chemistry (PANalytical MPD X-‘Pert Pro, Panalytical, Almelo, Netherlands). Samples were placed in the center of the three-dimensional analysis mount. Analysis was conducted using a 40 A, 45 kV x-ray gun from 30° to 90°, 18.87 ms dwelling time per step and 0.0001 degrees per step. One central location was analyzed per disc with two discs per group.

### 2.3. Assessment of cellular response

#### 2.3.1. Cell culture

Human female MSCs obtained after informed consent and isolated from adult bone marrow (Donor #8011L, Texas A&M Institute for Regenerative Medicine, College Station, TX) were cultured in MSC growth medium (GM) comprised of *α*MEM with 4 µM L-glutamine and 16.5% fetal bovine serum at 37 °C in 5% CO_2_ and 100% humidity and cultured to confluence in T75 flasks (Corning Inc., Oneonta, NY) before plating on the surfaces. For biological analysis, all four groups of surfaces were placed in a 24-well plate (Corning Inc.), and cells were plated at a density of 20 000 cells ml^−1^ at 0.5 ml per well. MSCs cultured on TCPS served as optical cell culture controls. Twenty-four hour after plating, GM were changed with subsequent media changes every 48 h after that for three, seven, nine and thirteen days. At each designated time point, cells were incubated for 24 h with fresh GM before harvest. Thus, cells designated as day 3 and day 7 were harvested on day 4 and 8. Cells designated as day 9 and 13 were harvested on day 10 and 14. Thus capturing the cell behavior for the full 24 h of the day of interest. These media were then defined as CM to indicate changes in the local environmental factors produced by the cells in the culture. CM were then collected from surfaces and stored at −80 °C, and MSCs were rinsed twice with 1× poshphate buffered saline (PBS), and placed in 0.5 ml of Triton-X100 and stored at −80 °C for biological assays.

#### 2.3.2. Factor production

Cell layers were lysed by ultrasonication at 40 V for 15 s well^−1^ (VCX 130; Vibra-Cell, Newtown, CT). The QuantiFluor* dsDNA system (Promega, Madison, WI) was used to determine total DNA content by fluorescence. Enzyme-linked immunosorbent assays were used to determine the levels of osteogenic and immunogenic factors in the CM. OCN (Thermo Fisher), osteoprotegerin (OPG; R&D Systems, Inc.), osteopontin (OPN; R&D Systems, Inc.), BMP2 and BMP4 (R&D Systems, Inc.), and interleukins 4, 6 and 10 (IL4, IL6, IL10; R&D Systems, Inc.) were quantified according to the manufacturer’s protocol.

### 2.4. Osteoinduction by local factors produced by MSCs in response to surface properties

All *in vivo* studies were carried out after approval from Virginia Commonwealth University’s Institutional Animal Care and Use Committee according to ARRIVE guidelines under protocol number AD10000675. Surgeries were conducted in two phases. Twenty-nine Crl:NU(NCr)-Foxn1nu mice, 8–9 weeks old, were purchased from Charles River (Wilmington, MA), 16 for the first phase of the study and 13 for the second phase. *In vivo* studies were conducted with an *n* = 8 implants per group based on a power analysis using an alpha of 0.05 and a power of 80% (delta = 5, sigma = 3, *m* = 1) to reveal a minimum of *n* = 7 per group for the study to yield statistical significance, 1 extra animal was included to ensure power if an animal reached a humane endpoint. Bilateral implants were placed, so the number of mice per experimental group was four to reduce the number of animals necessary for this study.

Phase 1 was performed to establish the system. We first assessed whether the *in vivo* assay was sensitive to DBM, which is a known osteoinduction agent (gift of LifeNet Health, Virginia Beach, VA). We also examined the osteoinduction ability of heat iDBM; lyophilized growth media, which included 10% fetal bovine serum (FBS) (GM); and lyophilized CM from MSCs cultured for 14 d on TCPS in GM. These media and cell controls were included to determine if agents present in the growth media, either as a function of the 10% FBS or as a result of the growth of MSCs on a non-Ti6Al4V surface, resulted in detectable bone formation in the mouse muscle pouch model. MSCs were cultured on TCPS at a seeding density of 10 000 cells cm^−2^ and GM were changed every 48 h. At day 14 [49–51], GM were changed and after 24 h, the now-CM were collected and stored at −80 °C. Growth media were incubated on TCPS without MSCs for 24 h to create the growth media control group.

In Phase 2, we tested the hypothesis that CM generated by MSCs on MMN^TM^ surfaces would support osteoinduction. Implant groups consisted of: (1) 20 mg iDBM, (2) 20 mg iDBM + 10ng of aqueous rhBMP2 (R&D Systems) added at the time of surgery in a concentration similar to the averaged *in vitro* production over 5 d of culture of MSCs on MMN^TM^ surfaces (MMN-CM concentration) [40, 52], and (3) 20 mg iDBM + 10 mg of lyophilized media from MSCs cultured on MMN^TM^ surfaces from day 5 through day 10 (D5–D10). For the rhBMP2 implant group, 10 µl PBSl containing 10 ng of rhBMP2 was added in the gel capsule and re-capped by the surgeon before placement into the muscle pouch.

#### 2.4.1. Preparation of implants

Based on the *in vitro* study comparing the response of MSCs to the three Ti6Al4V surfaces, we selected CM from cultures grown on MMN^TM^ substrates to assess osteoinductivity of CM generated by MSCs undergoing surface mediated differentiation in response to complex surface topographies. BMP2 production was elevated on all Ti6Al4V substrates on days 7, 10 and 14 compared to PEEK. We selected the MMN^TM^ surface for the *in vivo* assessment in part because BMP2 was highest in the day 14 MSCs cultured on MMN, but also because the pro-regenerative cytokines IL4 and IL10 were elevated whereas the pro-inflammatory IL6 was reduced.

Accordingly, MSCs were cultured on MMN^TM^ and TCPS at a seeding density of 10 000 cells cm^−2^. After plating, GM were changed at 24 h, and then every 48 h. Beginning at day 5, GM were changed daily through day 10 and each day’s CM were collected and stored at −80 °C. Media were pooled into a 50 ml conical tube and frozen at −80 °C prior to lyophilization. To lyophilize the media, the frozen media were uncapped, tightly covered with parafilm, and an 18-gauge needle was used to create holes in the layer in order to allow the evaporating liquid to escape the tube. The lyophilized proteins were then homogenized using weighing spatulas within a biological safety cabinet. Samples were measured and 10 mg of each group’s lyophilized powder was and placed into a UV sterilized size 5 gel capsule (Size 5, Torpac, Fairfield, NJ). Briefly, larger diameter halves of sterilized UV gel capsules were placed vertically in sterilized PCR tube holders and 20 mg of aDBM or iDBM was loaded into each half gel capsule massed by microbalance. All capsules were then capped with the smaller diameter half. All gel capsules and holders were sterilized again by UV light for 24 h in the biological safety cabinet (*n* = 8 implants/group). All groups were implanted on the day of surgery as described below in the mouse surgery section.

#### 2.4.2. Bilateral implantation of gel capsules into the muscle pouch of the gastrocnemius

Mice were acclimated for 5 d prior to surgery. Implants were randomized and implanted bilaterally into the hind limbs for *n* = 2 two per mouse. Each group consisted of *n* = 8 implants/group.

Anesthesia was induced with 4% isoflurane in 400 ml min^−1^ of oxygen until no response to toe pinch was observed. The surgical site was then prepared bilaterally using alternating washes of chlorhexidine and 70% isopropanol, repeated three times. Animals were then injected with 1 mg kg^−1^ of Sustained Release Buprenorphine LAB (Zoopharm, Laramie, Colorado) to provide 72 h of post-operative analgesia. Animals were transported to the procedure table and anesthesia was maintained with 2% isoflurane in 400 ml min^−1^ of oxygen, adjusting as necessary. A 1 cm longitudinal incision was made in the skin overlying the gastrocnemius muscle, exposing the muscle. A muscle pocket was formed between the gastrocnemius and the biceps femoris using blunt dissection. Care was used to gently dissect in order to maintain a bloodless field. A sterile, size 5 gelatin capsule loaded based on the group designation was inserted into this muscle pocket. The muscles and skin were then reapproximated and closed with 7 mm wound clips to avoid suture-related calcification. The same procedure was then conducted on the contralateral side. From induction to post-operative recovery, animals were kept euthermic by placing them on a water-circulated heating pad. Animals were closely observed the first 72 h following surgery and subsequently observed and weighed weekly. Wound clips were removed 14 d postoperatively. Thirty-five days post-operatively animals were killed via CO_2_ inhalation and the hindlimbs were harvested and stored in 10% buffered formalin [43, 53].

#### 2.4.3. Micro-CT analysis

Bone formation was assessed quantitatively by micro-CT using Dataviewer and Skyscan for scanning, imaging, and reconstructing to visualize and evaluate the new bone formation in the muscle pockets of the hind limbs. The legs were scanned in the 15 ml Eppendorf tubes packed with gauze above and below the leg to secure the sample in place. The tube was positioned so the long axis of the leg was perpendicular to the axis of the x-ray beam. All samples were calibrated under identical parameters at 50 kV and 150 µA, with 320 ms exposure time, at 17.9 µm magnified pixel size.

The micro-CT images were used to quantify new bone formation. The region of interest for bone quantification did not include the femur, tibia, or fibula. The mineralized bone tissue was differentiated from non-mineralized tissues using a fixed threshold of (70, 255). To determine the total bone volume of the new bone formation, the programs CTAn and CTvox (Bruker) were used to trace an area of interest by drawing a circular contour area per slice and quantifying the areas that remained white at the given threshold. With these areas summed over all the relevant slices, the bone volume was measured in mm^3^.

#### 2.4.4. Histological processing

Whole muscle was processed by Histion (Everett, WA) as described previously [53]. Briefly, samples were localized as needed using radiographic imaging on site. Samples were then decalcified in formic acid and subsequently embedded using a formalin-fixed paraffin-embedded tissue processing method. Samples were stained by hematoxylin and eosin (H&E) to assess the implant and muscle histology. One section was taken from the approximate center of the implant mass or as closely as possible to a trans-axial plane through the femur. Images of each sample were taken at 10X magnification using a light microscope (Carl Zeiss Meditec AG, Jena, Germany). Images were stitched and the lighting normalized across all stitched images boxes using Zen Pro.

#### 2.4.5. Histomorphometric analysis

Stitched images were used for histomorphometric tracing of demineralized bone chips and live bone analysis by a trained researcher using a bamboo drawing pen and image-J (NIH) to quantify each area, as described previously [53]. All areas of demineralized bone tissue were summated, and areas of live bone were summated, and percent live bone were calculated.

#### 2.4.6. Histological ranking

The histology images were ranked according to a scoring system adapted from ASTM2529-13 on a scale of 0 through 4 (standard guide for *in vivo* evaluation of osteoinductive potential for materials containing demineralized bone). Scores were determined by the percent new bone formation compared to total mineral tissue in the explants scored by histomorphometry. Samples were scored with 0 if no bone formation was calculated, 1 if there were less than 10% new bone-forming elements, 2 if there were between 10% and 20% bone-forming elements, 3 if there were between 21% and 30% new bone-forming elements, and 4 if greater than 30% bone-forming elements were present based on the histomorphometry analysis [43].

### 2.5. Statistical analysis

Data are means ± standard deviation for material characterization including optical profilometry, contact angle assessment, and XPS described in the methods. Data are means ± standard error mean of six independent cultures/variable for *in vitro; in vitro* cell experiments were repeated to ensure the validity of the results. Statistical analysis among groups at each time point was performed by one-way analysis of variance (ANOVA) and multiple comparisons between the groups were conducted with a two-tailed Tukey correction. Unpaired *t*-tests were used to compare analyses with only two groups and ANOVA for analyses with groups of 3 or more. Grubb’s outlier test was used to identify outliers. A *p*-value of less than 0.05 was considered statistically significant. All statistical analysis was performed with GraphPad Prism version 5.04.

## 3. Results

### 3.1. Evaluation of substrates

**Figure 1.**
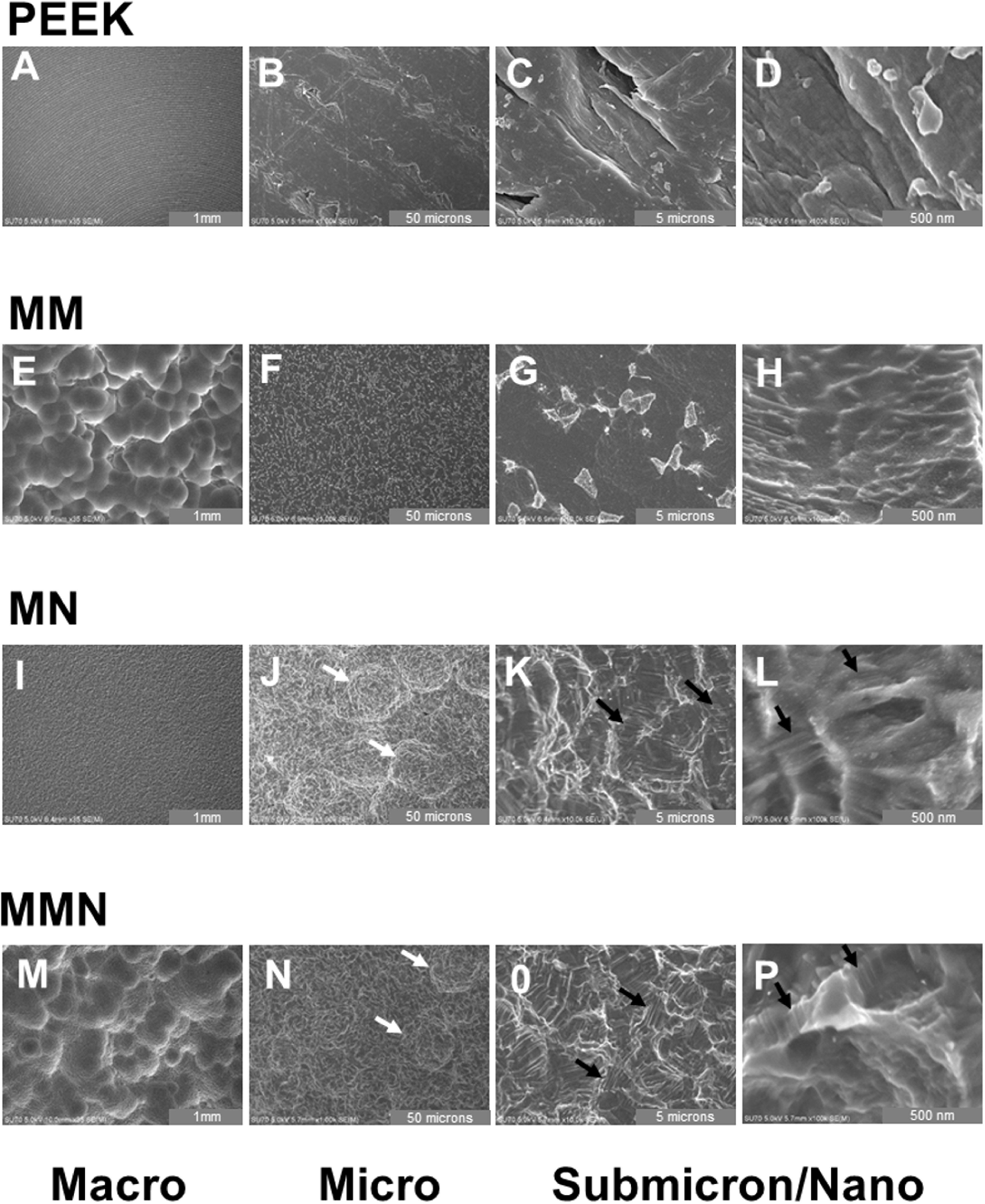
Scanning electron microscopy of PEEK and Ti6Al4V substrates possessing complex topography at the macro, micro, and meso/nanoscale. (LEFT) Macroscale SEM images captured at 35X. (MIDDLE) Microscale SEM images captured at 10 000X. (RIGHT) Nanoscale images were taken at 100 000X. Group names are: poly-ether-ether-ketone (PEEK), macro-/micro-rough (MM), micro-/nano-rough (MN), and macro-/micro-/nano-rough (MMN^TM^) Ti6Al4V surfaces. White arrows, microscale roughness generated by 2nd step acid-etching. Black arrows, multiscale micro-/nano-ridge structures generated by 2nd acid-etching step.

SEM of PEEK samples showed circular ridge formation during disc machining and increased magnification showed these micro ridges possessed microscale and submicron structure due to the machining processes. The end result was uneven gash and tear structures in concentric patterns (figures 1(A)–(C)). Grit-blasting and acid-etching the Ti6Al4V disks generated complex multiscale surface topographies at macro, micro, and nanoscale. MM and MMN^TM^ substrates possessed similar macroscale topography (figures 1(D) and (J)) with bubble-like morphologies seen at the macroscale. MN substrates possessed a similar texture at the macroscale imaging as the MMN^TM^ surface but lacked the circular cavities created by dual acid etching (figure 1(G)). These surface topographies at the microscale were comprised of smaller ridges and structures. However, MM substrates possessed only slight microstructures with heterogeneous topographies different from MN and MMN^TM^ surfaces across the surface (figure 1(E)). MMN^TM^ and MN possessed similar micron scale morphologies (figures 1(H) and (K)). All groups had dissimilar nanoscale topography, but MMN^TM^ and MN had similar micro-/nano-scale ridge formations (figures 1(F), (I) and (L)) dispersed across the substrate surfaces.

**Figure 2.**
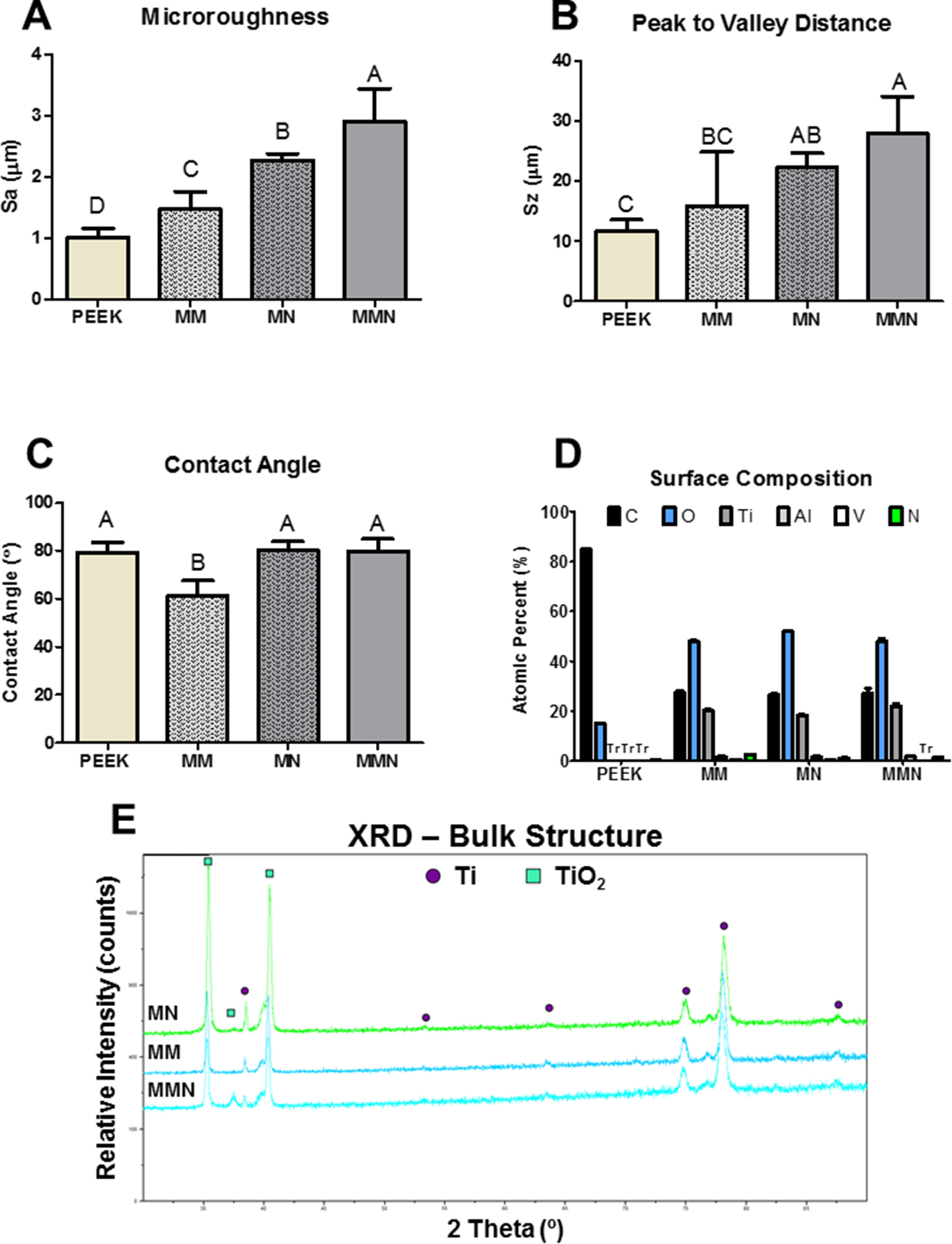
(A) Average areal microroughness and (B) average peak to valley distance for the 4 substrates using optical profilometry. (C) Quantification of surface wettability by sessile drop contact angle measurement. Groups not sharing letters are significantly different at *p* < 0.05. Groups not sharing letters are significantly different at *p* < 0.05 (D) semi-quantitative x-ray photoelectron spectroscopy analysis of surface elemental composition, and (E) x-ray diffraction profiles of the surface and bulk chemistry of the Ti6Al4V substrates. Tr is trace amounts of each element were detected by XPS of bulk structure and circles are titanium (Ti) whereas squares are predicted to be titania (TiO_2_) based on elemental reference standards for EDX [88]. Group names are poly-ether-ether-ketone (PEEK), macro-/micro-rough (MM), micro-/nano-rough (MN), and macro-/micro-/nano-rough (MMN^TM^) Ti6Al4V surfaces.

Reflective optical profilometry showed MM substrates had increased average microroughness compared to PEEK and processing to generate nanostructure formation further increased average microroughness on MN and MMN^TM^ substrates in a macroscale topography dependent manner (figure 2(A)). MMN^TM^ was significantly higher than MM and PEEK peak-to-valley height; MMN^TM^ and MN substrates were similar, and MN and MM substrates were also similar. MN was significantly higher than PEEK where MM and PEEK did not have differences in peak-to-valley height (figure 2(B)).

Surface wettability analysis showed PEEK substrates were less wettable compared to MM Ti6Al4V substrates. Nanostructure formation of the Ti6Al4V substrates also reduced the wettability of MN and MMN^TM^ substrates to the same level found on PEEK (figure 2(C)). All substrates demonstrated intermediate wettability independent of surface processing. Surface chemistry was characterized by XPS. XPS analysis showed carbon and oxygen content matching the chemical formula for PEEK polymer. All Ti6Al4V groups had similar concentrations of oxygen, carbon, titanium, aluminum, and vanadium (figure 2(D)). XRD profiles were then taken for the Ti6Al4V substrates. Profiles for each group (MM, MN, and MMN^TM^) showed similar peak location in the bulk of the titanium alloy, MMN^TM^ substrates contained an additional peak 37°, which is most likely TiO_2_ (figure 2(E)).

### 3.2. Cellular response to surface texture

**Figure 3.**
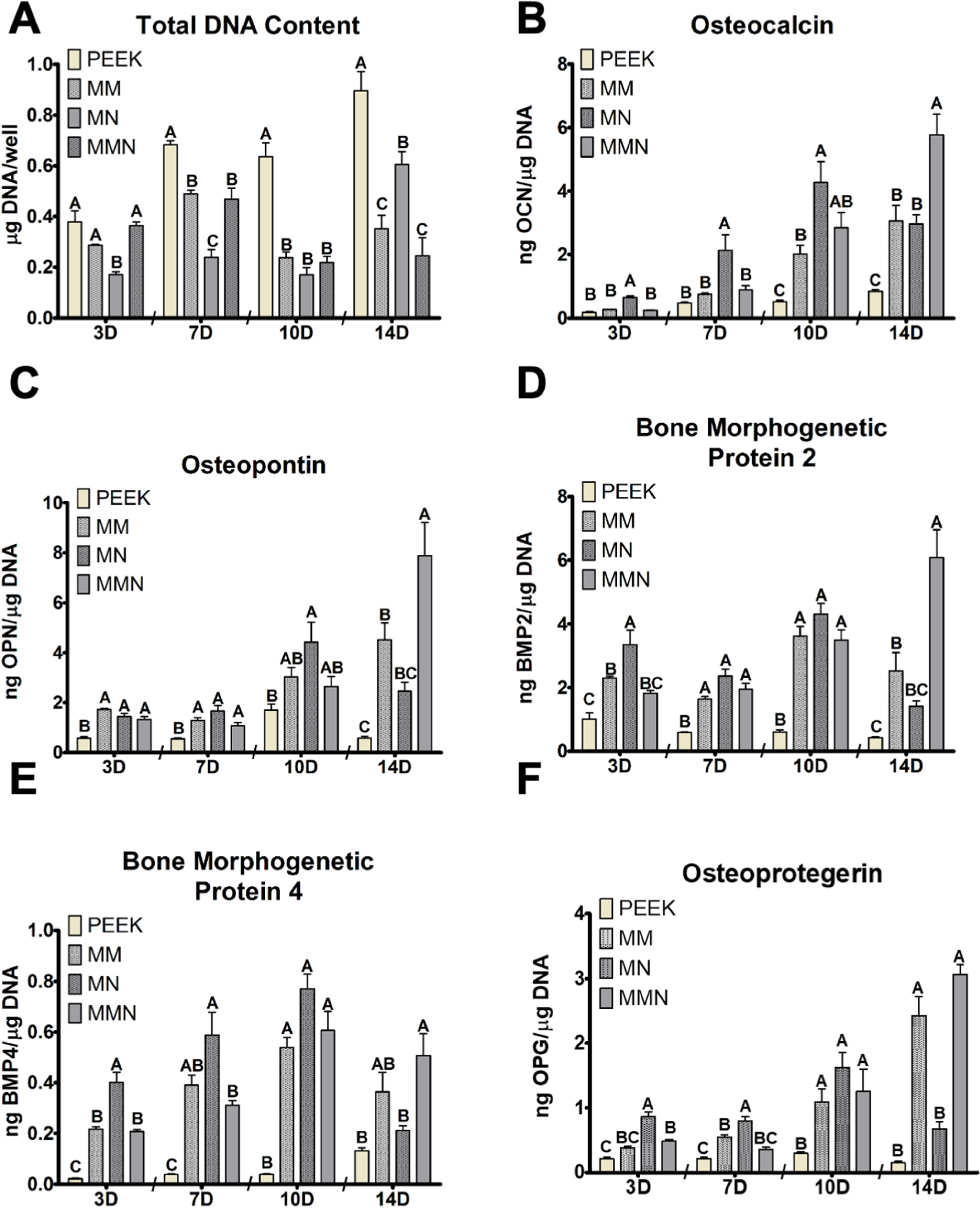
Cellular response to PEEK and Ti6Al4V surfaces. (A) Total DNA content was determined in the cell layer lysate. (B) Osteocalcin, (C) osteopontin, (D) bone morphogenetic protein 2 and (E) BMP4, (F) osteoprotegerin were determined in the conditioned media at days 4 (designated D3), 8 (designated D7), 10 and 14. Groups not sharing letters are significantly different at *p* < 0.05 within each time point between surfaces. Group names are poly-ether-ether-ketone (PEEK), macro/micro-rough (MM), micro/meso/nano-rough (MN), and macro/micro/meso/nano-rough (MMN^TM^) Ti6Al4V surfaces.

Total DNA content at day 3 showed less MSC DNA on MN surfaces. At day 7, all Ti6Al4V groups had decreased total DNA content compared to PEEK, and MN was further significantly decreased. However, by day 10, Ti6Al4V substrates possessing macroscale roughness (MM and MMN^TM^) had the same total DNA content as MN and all Ti6Al4V groups were less than PEEK. At day 14, PEEK had the highest DNA content, followed by MN, and the lowest total DNA content was on microroughened surfaces, MM and MMN^TM^ (figure 3(A)).

OCN and OPN were determined in the CM to assess osteoblastic differentiation of MSCs. OCN increased temporally on Ti6Al4V substrates. At days 3 and 7 MSCs produced low amounts of OCN but it was increased on MN (figure 3(B)). OCN was increased on the Ti6Al4V surfaces at day 10; the nano-modified groups produced the highest concentration of OCN and MM groups produced the same as MMN^TM^. MMN^TM^ produced the most OCN at day 14 and MM and MN substrates produced similar levels (figure 3(B)). OPN was increased on Ti6Al4V substrates at day 3 and 7, compared to PEEK and surface morphology did not change OPN production. MN was increased compared to PEEK at day 10, while MM and MMN^TM^ were not. At day 14, MSCs produced more OPN on MMN^TM^ surfaces compared to MM and MN, and MM substrate MSCs produced more OPN than PEEK (figure 3(C)).

Paracrine signaling factors BMP2, BMP4, and OPG were measured in the CM to evaluate how MSCs were altering the microenvironment at the surface. BMP2 was increased at day 3 on MN, compared to MM and MMN^TM^, and MM was increased compared to PEEK. However, all Ti6Al4V groups were increased compared to PEEK at day 7 and day 10. MMN^TM^ produced the greatest amount of BMP2 on day 14 and MM and MMN^TM^ were similar, while MN and PEEK were similar (figure 3(D)). BMP4 responded similarly; however, at day 3 and 7 MM and MMN^TM^ were increased compared to PEEK and MM and MN were not different from each other at day 7. All Ti6Al4V groups were increased compared to machined PEEK at day 10, and MM and MMN^TM^ both produced similar amounts of BMP4 at day 14 and MN and PEEK were not different (figure 3(E)). OPG was increased on nanomodified Ti6Al4V surfaces at day 3 and was further increased on MN compared to the other Ti6Al4V surfaces. At day 7, MN produced the most, followed by MM, and MMN^TM^ was not different from PEEK. However, 3 d later at day 10 OPG was increased on the Ti6Al4V surfaces compared to PEEK. Macroroughened Ti6Al4V groups were increased on day 14, and MN was not different from PEEK (figure 3(F)).

### 3.3. Immuno-regulation by soluble cytokines

**Figure 4.**
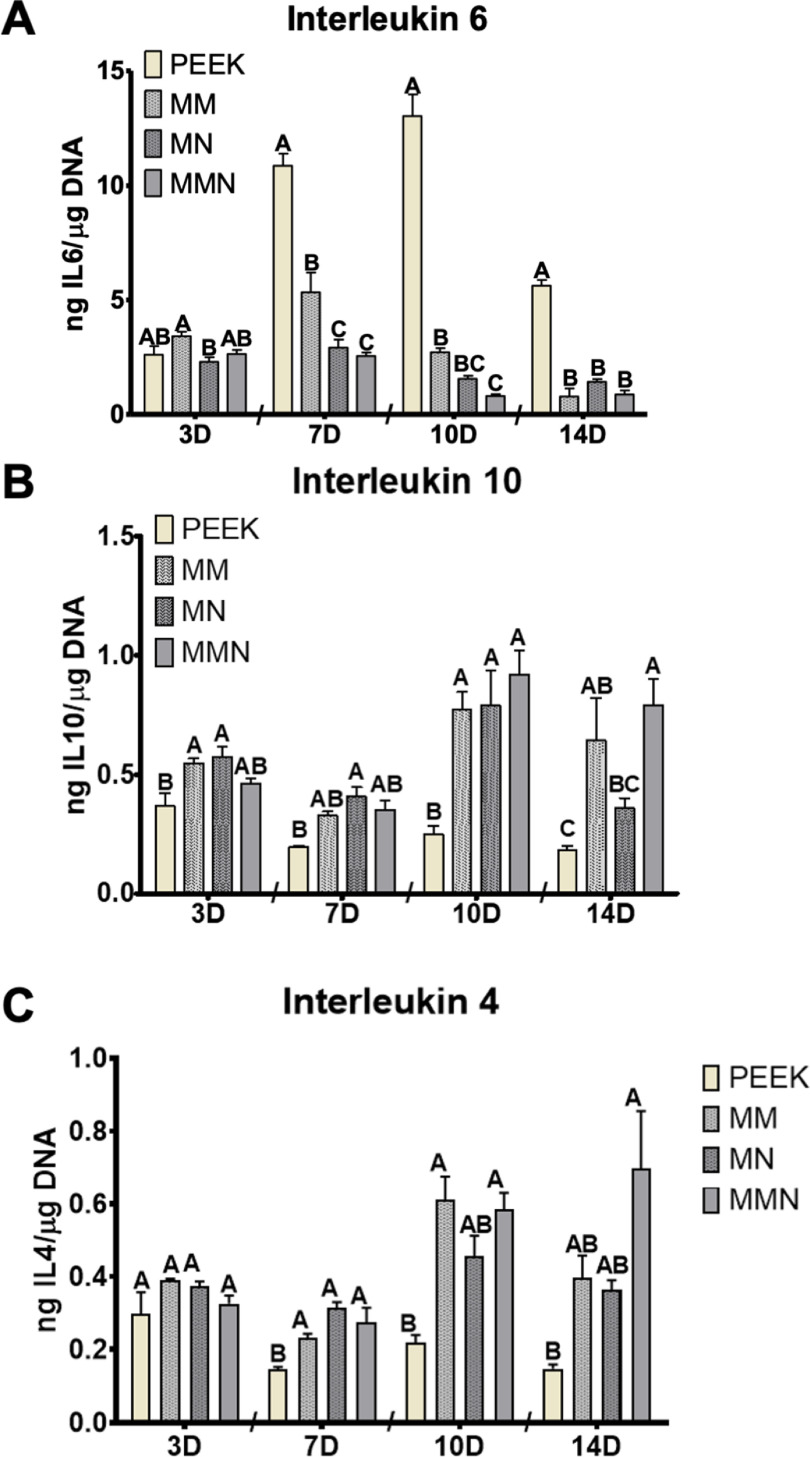
Cellular response to PEEK and Ti6Al4V surfaces. (A) Interleukin 6, (B) interleukin 4, and (C) interleukin 10 were determined in the conditioned media at days 4 (designated 3D), 8 (designated 7D), 10 and 14. Groups not sharing letters are significantly different at *p* < 0.05 within each time point between surfaces. Group names are poly-ether-ether-ketone (PEEK), macro/micro-rough (MM), micro/meso/nano-rough (MN), and macro/micro/meso/nano-rough (MMN^TM^) Ti6Al4V surfaces.

Production of the proinflammatory IL6 and anti-inflammatory IL4 and IL10 was assessed over the 14 d. IL6 was higher on MM surfaces compared to MN at day 3. However, at days 7, 10, and 14 the amount of IL6 produced by MSCs was higher on PEEK substrates compared to all Ti6Al4V surfaces, and at days 7 and 10 the presence of nanostructures further decreased IL6 production (figure 4(A)). IL4 and IL10 were not altered significantly at day 3, only MM and MN cultures had increased IL4 and IL10 compared to PEEK at day 3. IL4 was increased on Ti6Al4V surfaces at day 7, while MN was the only group producing more IL10 than PEEK at day 7. Both IL4 and IL10 were increased on microroughened surfaces at day 10 and PEEK and MN were not different from each other for IL4. At day 14, MMN^TM^ produced the highest concentration of IL4 and IL10, but was not different than MM group for IL4. MM was not different from MN or PEEK for IL4, but was different from PEEK for IL10 (figures 4(B) and (C)).

### 3.4. Mineralization in an *in vivo* model of ectopic osteogenesis

**Figure 5.**
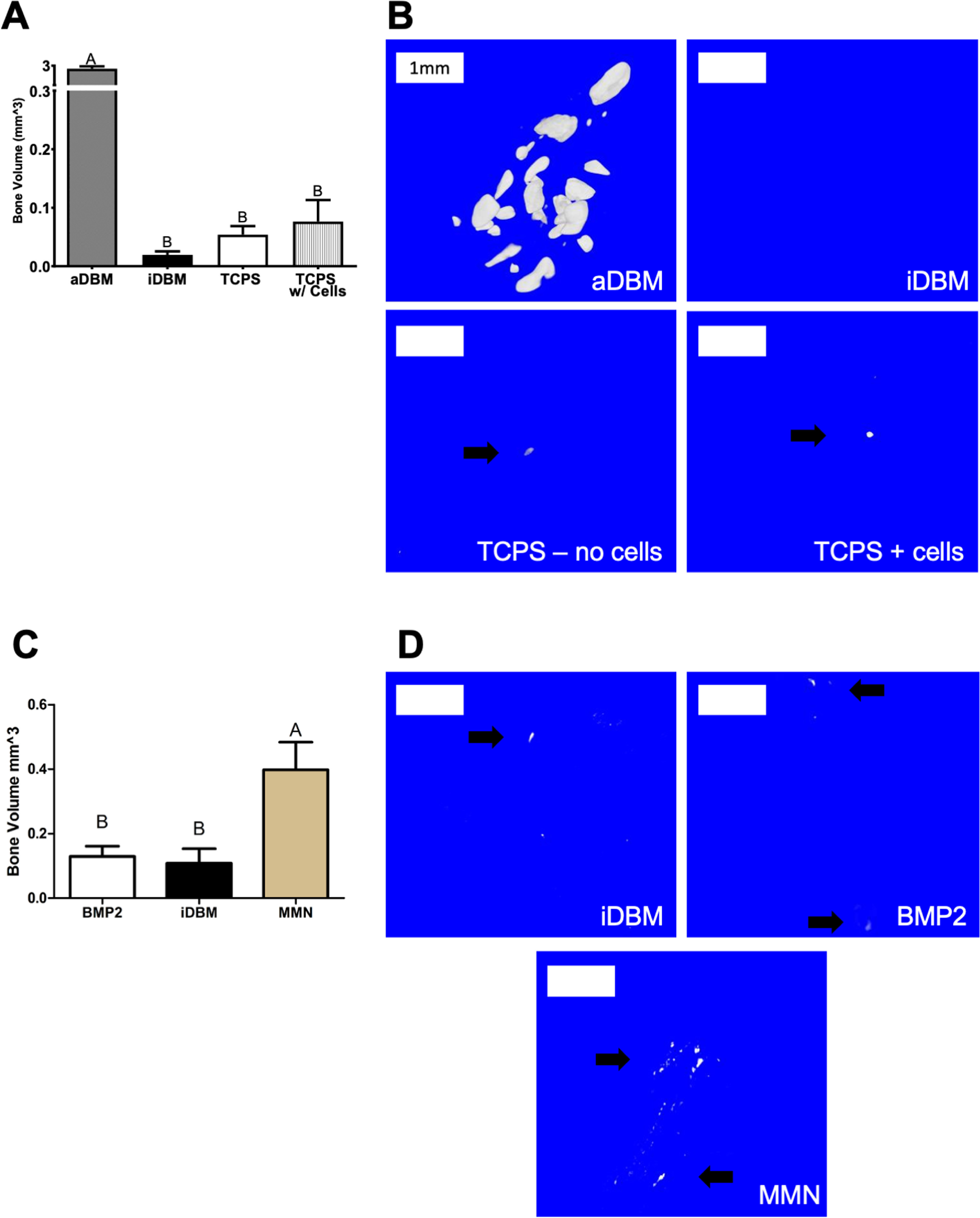
Ectopic models of osteogenesis in athymic nude mice gastrocnemius. Phase 1: (A) micro-CT quantification of mineralized tissue within the muscle pouch of the gastrocnemius for active DBM (aDBM), heat inactivated DBM (iDBM), iDBM supplemented with lyophilized growth media (TCPS-no cells), and iDBM supplemented with lyophilized conditioned media from MSCs cultured on TCPS for 14 d (TCPS + cells). (B) Representative micrographs of the four groups. Phase 2: (C) micro-CT quantification of mineralized tissue within the muscle pouch of the gastrocnemius for iDBM, iDBM supplemented with 10 ng of rhBMP2, and iDBM supplemented with lyophilized conditioned media collected from MSCs cultured on MMN^TM^ surfaces beginning on day 5 through day 10 of culture and pooled. (D) Representative micrographs of the three groups. Arrows are markers of mineralized tissue formation within the region of interest of the micro-CT scans. Groups not sharing letters are significantly different at *p* < 0.05. White boxes are scale bars at 1 mm.

In order to evaluate the effect of paracrine signaling factors secreted by cells in contact with MMN^TM^ Ti alloy surfaces, we first validated the ASTM *in vivo* assay for osteoinductivity. At the micro-CT level, active DBM demonstrated robust mineralization throughout the implanted tissue (BV ∼ 3 mm^3^) as a positive control. Furthermore, iDBM and iDBM supplemented with lyophilized media and CM from MSCs cultured on TCPS showed little mineralization as shown by the micro-CT reconstructions (figure 5(A)) and quantification (figure 5(B)). These results demonstrate that MSCs cultured on TCPS, and media alone are negative controls and incapable of osteoinduction in the standard assay and show that the system can identify ectopic osteogenesis if it is present.

**Figure 6.**
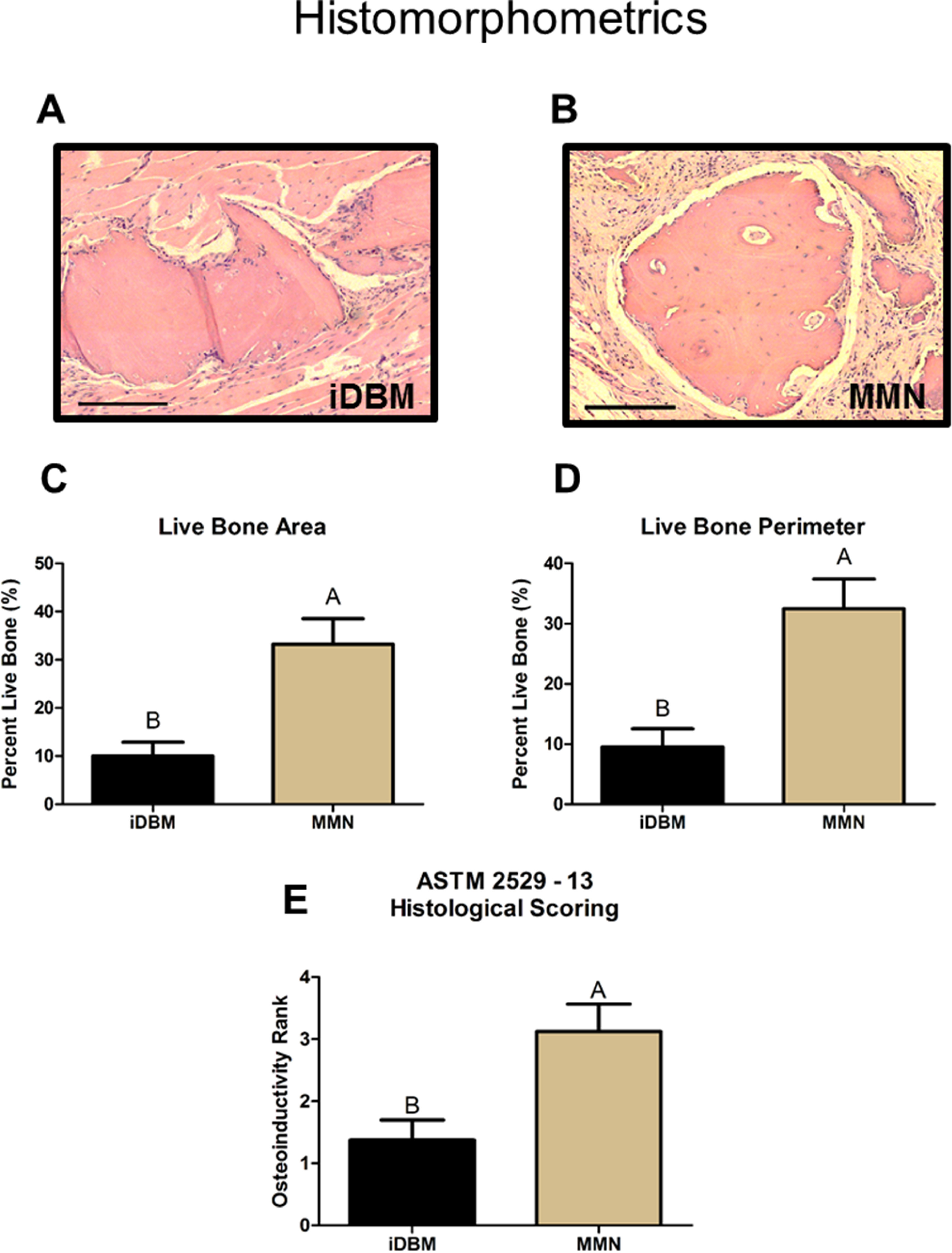
Histological analysis of new bone formation ectopically. (A) and (B) Representative histological images of the H&E staining for both the (A) iDBM and the (B) MMN^TM^ groups. (C) Quantification of the live bone area using quantitative histomorphometry. (D) Quantification of the live bone perimeter using quantitative histomorphometry. (E) Ranked scoring of each sample according to an adapted ASTM Standard 2529–13 (standard guide for *in vivo* evaluation of osteoinductive potential for materials containing demineralized bone). Black arrows mark areas of live bone with cells populating lacunae. Black lines are scale bars at 1 mm. Groups not sharing letters are significantly different at *p* < 0.05.

We then evaluated the effect of iDBM supplemented with rhBMP2 at a similar level produced by the cells in culture (rhBMP2; 10 ng, R&D Systems) or iDBM supplemented with 10 mg lyophilized media from MSCs cultured on MMN^TM^ Ti alloy substrates [40, 53]. As expected with the replicated negative control, iDBM alone was not osteoinductive, nor was iDBM supplemented with 10 ng BMP2. In contrast, iDBM plus CM from MMN^TM^ implants demonstrated increased bone volume compared to both BMP2 and iDBM groups (figures 5(C) and (D)) as quantified by micro-CT. To further evaluate this effect, MMN^TM^ and iDBM groups were processed histologically with H&E staining for quantitative histomorphometry to determine new bone formation. These data showed increased live bone area (figure 6(C)) and live bone perimeter in MMN^TM^ samples compared to iDBM (figure 6(D)). Histological slices of each group showed greater areas of cell-populated canaliculi in bone pieces from the MMN^TM^ group (figures 6(A) and (B)). Furthermore, histological samples were ranked according to ASTM 2529–13 to determine osteogenic activity of DBM lots. iDBM had a new bone formation percentage of 9.9% and an average rank of 1.375 (table 1), failing the osteogenic ranking assessment. MMN^TM^ samples demonstrated an increased averaged new bone percentage of 31.8% and an average histological ranking of 3.125 (table 1), passing the assessment of osteogenic activity according to the standard.

**Table 1.** Histological ranking of samples for inactive demineralized bone matrix (iDBM) and MMN^TM^ conditioned media DBM (MMN^TM^). Histological rank 0–4 were scored based on new bone formation adapted from ASTM2529-13 standard for osteogenesis of DBM batches. Each independent leg is a sample. Ranking less than 2 is a failing batch for osteoinductivity.

	iDBM rank	MMN rank
Sample 1	1	4
Sample 2	3	4
Sample 3	2	4
Sample 4	1	1
Sample 5	1	2
Sample 6	2	4
Sample 7	1	4
Sample 8	0	2
Average	1.375	3.125

## 4. Discussion

The present data demonstrate that Ti6Al4V surfaces possessing a combination of complex topographies like macroroughness, microtopography, and nanoscale feature formation temporally regulate the production of osteogenic factors over time. Surfaces possessing a subset of these complex structures are also capable of inducing osteogenic differentiation of MSCs into osteoblasts and the rate of this differentiation and regulation of local factors is influenced by the surface properties. Moreover, paracrine signaling factors produced by cells cultured on substrates comprising complex multiscale biomimetic topographies at the macro/micro/meso/nanoscale can induce bone formation. This study proves that the microenvironment—as created by cells on these surfaces in an *in vitro* culture—is osteoinductive in a standard ectopic model of osteogenesis. This finding supports further hypotheses that an osteoinductive microenvironment during implantation may improve osseointegration with new bone formation.

Surface properties have long been known to be important for the integration of Ti-based implants. Substrates created by machining and processed by grit-blasting and acid-etching result in Ti6Al4V surfaces possessing microscale roughness that have demonstrated success clinically in the field of periodontology and implant dentistry [24, 54–56]. In the present study, further processing of roughened surfaces using proprietary grit-blasting and acid-etching create additional microstructure and nanotexture formation; and reduce surface wettability in a manner similar to previous studies [33, 57]. However, in this study we have demonstrated a surface processing method that creates a similar evenly dispersed, heterogenous nano-topography regardless of the starting macroroughness of the implant surface, as seen in the SEM images of both MN and MMN^TM^ surfaces.

PEEK implant electron micrographs show complex tear morphology due to the machining process and confirms the roughness quantified by optical profilometry. However, these surface properties were not sufficient to generate an osteogenic response, likely governed by differences in bulk chemistry and limited multiscale topography.

Macroscale electron micrographs show similar morphology between MM and MMN^TM^ surfaces that confirms the same processing treatment prior to modification at the micron and nanoscale. The macroscale surface modification generated by dual acid etching resulted in similar reductions in DNA content by day 7 and increased OCN and OPG. This was correlated with early increases in BMP2 and BMP4. Addition of micro and nanotopography to either the macro-textured surface or a machined surface, caused a further increase in BMP production, which was sustained through day 14 of culture on the MMN^TM^ surface. These results confirm the importance of the biomimetic multiscale topography to attain the full osteogenic panoply of outcomes.

These results also demonstrate the importance of assessing surface properties using multiple parameters. Implant modifications can induce surface morphologies that vary significantly from each other but have matching quantifiable surface roughness [54, 58–60]. The use of scanning electron micrographs and qualification of surfaces at the macro-, micro-, and nanoscale provides important predictive information on potential effectiveness of surface treatment on altering cellular response.

Biologically, nanostructures have been shown to alter cell proliferation and attachment and macroscale roughness affects production of cytokines over a longer period [13, 25, 27, 29, 61–63]. Ti6Al4V surfaces possess superior material properties, such as wear resistance, and alloy crystalline phases that allow tuning for physical morphologies that closely resemble the biological surface of native bone. The result is cellular phenotypes with reduced production of proinflammatory IL6 signaling during surface-mediated osteogenesis and increased production of pro-regeneration cytokines like IL4 and IL10 that have been shown to increase implant retention [61, 63–65]. Newer polymeric materials for orthopedic and dental applications like PEEK, have mechanical moduli that are more similar to cortical bone but have also been shown to increase inflammatory profiles in preclinical and basic research studies [65–67].

Wettability is another key regulator of cellular response to a biomaterial. In this study, while all substrates possess intermediate wettability, the microroughened Ti6Al4V substrates had increased surface wettability compared to PEEK polymers. Moreover, nano feature formation decreased the wettability of Ti6Al4V surfaces, suggesting the nanofeatures alter the interaction of liquids on the implant surface. This is unsurprising: surface roughness and the formation of nanostructures have been shown to directly alter the contact angle and surface wettability of surfaces including Ti alloys [64, 68].

*In vitro* surface wettability has been studied with respect to osteoblast adhesion and behavior on dental implant surfaces. Increasing surface wettability and creating hydrophilic implant surfaces have been shown to alter protein adsorption [69, 70], cell attachment [20, 71], and production of important paracrine signaling molecules that modulate immune response to an implanted material [66, 72]. Although surface processing reduced overall wettability of the surface, the cellular response was still enhanced, suggesting that topography plays a larger role in longer term cellular response. Additional further processing of MMN^TM^ and MN surfaces to increase surface wettability may lead to increased cellular response during surface mediated osteogenesis. MSCs are sensitive to surface topography both at the microscale and to submicron surface textures [73]. MSC response to these four surfaces showed a phenotypic decrease in cellular proliferation on Ti6Al4V surfaces compared to PEEK over 14 d. Moreover, these cells were also sensitive to small morphological changes by delaying proliferation on only MN modified surfaces. The presence of macroscale roughness on the implant surface did decrease proliferation compared to PEEK, but slight proliferation still occurred until day 14. These effects could be attributed to differences in surface area as well as to alterations in adsorbed proteins on the substrates. These binding epitopes are then sensed by MSC receptors on the implant surface and previous literature has shown that activation of these receptors can lead to reduced proliferation as maturation occurs [31, 37, 74].

Osteoblast differentiation is inversely related to proliferation; thus, peak production of factors associated with osteogenesis will occur at different times depending on the substrate surface properties, like topography or wettability. This physiologic balance and temporal regulation of proliferation vs differentiation of progenitor cells is important to ensure sufficient cell population within the implant micro-environment while also promote regeneration [1, 75]. Osteoblast maturation is jointly regulated by macroscale topographies, microscale roughness and nanotexture formation [25, 39]. Furthermore, MSCs still differentiate into osteoblasts in a surface-mediated manner in growth media despite lacking any exogenous media supplements found in osteogenic media. This observation is supported by the increasing production of both OCN and OPN as well as paracrine signaling factors BMP2, BMP4, and OPG. MSCs on machined PEEK do not undergo surface-mediated differentiation into osteoblasts demonstrated by low levels of both OCN and OPN. These data suggest that cells sense the various types of surface chemistry, surface modifications, and the combination of modifications have additive effects on cellular response. These considerations are important preclinically as recent reports have shown variability in personalized responses to implant surfaces possessing microroughness alone as well as sex differences in regenerative response during culture or after implant placement [76, 77]. Therefore, implant surfaces that possess a hierarchy of topography may facilitate osteogenesis throughout the integration process.

Interleukins were quantified to determine how MSCs were regulating immune lineage cells. Proinflammatory IL6 was upregulated on PEEK surfaces compared with all titanium surfaces at 7 d and remained upregulated thereafter. Small levels of IL6 have been shown to be important to regulate cellular migration around an implant [78]. However, prolonged elevated levels of IL6 can result in a cytotoxic microenvironment and *in vivo* can result in fibrous tissue formation and implant failure. These results are similar to published clinical data showing PEEK implants may result in nonunion or subsidence [14, 61].

Anti-inflammatory IL4 and IL10 were increased on Ti6Al4V substrates compared with PEEK with the greatest trends in upregulate at 10 and 14 d. Both of these anti-inflammatory cytokines have been investigated previously in the immune system and are increased in a microroughness and hydrophilic manner [79, 80]. MSCs have been shown to exert immunomodulatory signals and it is likely, in this system, that MSCs on microroughened and nanotextured surfaces increase production of IL4 and IL10, which polarizes macrophages into a regenerative M2 response [63, 79–82]. Previous studies using similar surfaces have demonstrated distinct differences between bulk chemical structure and overall cellular response. PEEK has been shown to increase pro-inflammatory cytokines and reduction in pro-regenerative cytokines [65]. These distinct differences have also been correlated in the clinic with increased rates of subsidence after fusion [10, 14, 17, 83–85].

Previous studies have used CM or trans-well co-cultures *in vitro* to assess paracrine signaling of soluble factors necessary for osteogenesis. These studies show that surfaces possessing microroughness are able to initiate the production of soluble signaling factors into the peri-implant microenvironment [40, 41]. The hypothesis that factors produced by cells in contact with the surface are important clinically is supported by the observation that in periodontology, micro/meso/nano-textured surfaces applied to threaded endosseous implants have demonstrated strong clinical success with increased implant retention rates and reduced implant failures [86].

*In vitro* studies are often limited with one cell type on the surface acting as the signal to osteoprogenitor cells, rather than the more complex collection of cells present *in vivo* and the temporal regulation of local factors from these varied cell populations. The mouse muscle model for assessing osteoinductivity enabled the capsule-based implantation of a combination of soluble factors secreted by osteoprogenitor cells. This usage further enabled the observation that the microenvironment with these factors signaled other cell types to produce new bone ectopically *in vivo*. Our previous observation, that inhibiting the action of BMP2 blocked the surface effect *in vitro* [40], suggested, unsurprisingly, that BMP2 is one important factor responsible for the osteoinduction observed *in vivo*. The observation, however, that iDBM + BMP2 was not osteoinductive in the present study, indicated that BMP2 is not the only constituent of the microenvironment, the CM generated by MSCs on MNN™ surfaces, that is playing a role.

There are several reasons that iDBM + BMP2 was not effective. The concentration of BMP2 in the CM was much lower than is used clinically [87], and may not have been sufficient to induce osteoinduction under the conditions of the study. We used BMP2 purchased from a supplier other than BMP2 commonly used clinically. Finally, the delivery system with iDBM and the carrier with a gel capsule is not the delivery system that is used clinically.

In this study, Ti6Al4V surfaces that possess the multiscale biomimetic structures at the macro, micro, meso and nanoscale stimulate MSCs to generate factors that support bone formation ectopically in a mouse model of osteoinduction. Ectopic bone formation is the gold standard to determine the effectiveness of an implanted biomaterial with regards to support de novo osteogenesis *in vivo* and ASTM 2529–13 is the active standard for evaluating materials containing DBM for osteoinductive potential [43]. In many cases, the use of this standard is limited because the actual material cannot be implanted in rodent muscle. Instead, we focused on the chemical environment generated by MSCs cultured on the MMN^TM^ surface. Additionally, inflammatory regulation is an important factor in development of new bone *in vivo*, but it was necessary to use athymic nude mice as the CM were generated by human MSCs, which is also a potential limitation as these mice lack a competent fully developed immune system seen in other preclinical models of implantation into bone.

Even with these limitations, we present compelling data demonstrating that MSCs cultured on biomimetic Ti6Al4V substrates generate a microenvironment that is osteoinductive in this ASTM standard assay. Cell culture media alone and CM from MSCs cultured on tissue culture plastic for 14 d are unable to stimulate bone formation ectopically. These samples lack the soluble signaling factors necessary for mineralization to occur. In contrast, the addition of lyophilized CM from MSCs differentiating in response to MMN^TM^ surface properties from day 5 to day 10 of culture possess the combination of factors to stimulate bone formation ectopically. Moreover, when BMP2 is supplemented into iDBM, at *in vitro* concentrations similar to MSCs cultured in contact with the MMN^TM^ surface, osteoinductivity does not occur suggesting it is a combination of multiple soluble factors that are required for osteogenesis.

Measuring the basic biological response to implant surfaces is clinically important to support common methods to restore function and quality of life for patients [1, 2]. For treatment of chronic back pain, loss of teeth, or trauma, to name a few, dental and orthopedic implants must maintain stability to restore function. Physicians in these fields currently rely on the structural stability of titanium or PEEK materials and in challenging scenarios, they may consider options to further facilitate bone formation by using exogenous sources of bone inductive proteins like rhBMP2. That is, the clinician considers, not only the material itself, but the local peri-implant microenvironment and cellular signaling needed to support bone formation as well. This study suggests that a titanium material with a biomimetic surface can contribute to the cellular response and an osteoinductive microenvironment that facilitates new bone formation. This finding elucidates new approaches and important factors to improve implant fixation and patient quality of life especially those suffering compromised bone qualities [59]. The development of biomimetic implant surface topographies has been shown to increase osteogenesis and cellular differentiation of progenitor cells, and we continue to assess how these effects can support treatment planning for physicians.

## 5. Conclusions

Collectively, these data show that MSCs are sensitive to a combination of titanium implant surface properties including macro and microscale roughness and nanoscale feature formation. In the *in vitro* portion of this study, MSCs cultured on the MM, MN, and MMN^TM^ substrates produced a positive combination of factors compared to controls. In the *in vivo* portion of this study, new bone formation was sensitive to the combination of factors observed in various culture media. With an ectopic model of osteoinduction, this study found that the combination of osteogenic soluble signaling factors, observed in the CM from MMN™ substrates and implanted with a gel carrier into muscle, created an osteoinductive microenvironment.

## Data Availability

The data that support the findings of this study are available upon reasonable request from the authors.
